# The relationship between dietary patterns and overweight and obesity among adult in Jiangsu Province of China: a structural equation model

**DOI:** 10.1186/s12889-021-11341-3

**Published:** 2021-06-25

**Authors:** Yuan-yuan Wang, Ting Tian, Da Pan, Jing-xian Zhang, Wei Xie, Shao-kang Wang, Hui Xia, Yue Dai, Guiju Sun

**Affiliations:** 1 Key Laboratory of Environmental Medicine and Engineering of Ministry of Education, and Department of Nutrition and Food Hygiene, School of Public Health, Southeast University, 87 Ding Jia Qiao Road, Nanjing, 210009 China; 2 Institute of Food Safety and Assessment, Jiangsu Provincial Center for Disease Control and Prevention, No.172 Jiangsu Road, Nanjing, 210009 Jiangsu China

**Keywords:** Dietary pattern, Structural equation modelling, Overweight and obesity, China

## Abstract

**Aims:**

This study aimed to analyze the relationship between diet and overweight and obesity in Jiangsu Province by using structural equation modeling (SEM), and to determine dietary differences between genders in the model.

**Methods:**

Data from 1739 individuals (53.8% female, *n* = 935) were analyzed. Exploratory factor analysis (EFA) and confirmatory factor analysis (CFA) were used to classify dietary patterns. SEM and multivariate logistic regression were used to explore the relationship between dietary patterns and overweight and obesity.

**Results:**

Overweight and obesity was found in 49.1%, and no difference was found in gender (51.2% of men and 47.2% of women, respectively; *P* = 0.090). Three dietary patterns: the traditional dietary pattern (i.e., poultry, light-colored vegetables, red meat and its products, cereals and tubers products, condiment, oils and dark-colored vegetables), the fruit-egg dietary pattern (i.e., fruit, whole grains, pickled vegetables and eggs and eggs products) and nut-wine dietary pattern (i.e., nut, wine and pastry snacks) were established by using EFA and CFA. It was found that the traditional dietary pattern for adult male was positively associated with the overweight and obesity in Jiangsu Province of China through multivariate logistic regression and SEM (OR = 1.954; 95%CI: 1.258 ~ 3.036; β =0.121, *P* < 0.05, respectively).

**Conclusion:**

The traditional dietary pattern only have positive association with overweight and obesity in men in Jiangsu Province, China.

## Introduction

According to the World Health Organization (WHO), overweight and obesity are increasing globally, which has become the fifth leading cause of death worldwide [1]. The similar issue has become more and more prominent in China. According to the Report on the Status of Nutrition and Chronic Diseases of Chinese residents (2015), the rates of overweight and obesity for residents over 18 years old were 30.1 and 11.9%, respectively [2]. The constantly growing trend of overweight and obesity are taking us further away from the global goal of eradicating obesity — we seem to be losing the war against obesity [3].

Obesity is a complicated multifactorial chronic disease, which has an important contribution to the global incidence of cardiovascular disease, hypertension, type 2 diabetes mellitus and cancer [4–6]. Recently, several studies showed that individuals who were obese might be more likely to get COVID-19 [7, 8]. Overweight and obesity are caused by the interaction between genetics, environment and human behavior [9]. Diet structure in human behavior has been proved to be the independently risk factor of overweight and obesity [10]. In recent years, compared with traditional dietary analysis (simply focused on individual nutrients or foods), the analysis of dietary patterns has emerged as a comprehensive approach [11]. However, few studies tested the rationality of the dietary patterns obtained. And to our knowledge, no studies explained the relationship among different dietary patterns and the direct and indirect associations with overweight and obesity and socio-demographic and diet intake in Jiangsu Province of China. Fortunately, Structural equation modelling (SEM) is regarded as a suitable statistical method, which combines the methods of factor analysis and path analysis to test the validity of dietary patterns and to figure out the direct and indirect relationship between potential variables and observation variables [12, 13]. Errors and individual differences are considered in SEM [14].

Therefore, the purpose of this study was to analyze the relationship between diet and overweight and obesity in adult in Jiangsu Province of China by using structural equation modeling, and to explore the gender difference in diet.

## Participants and methods

### Study population

This study was the second phase of an observational population-based prospective nutrition and health study in 2014 in Jiangsu Province, China. The first phase was built in 2007. A multi-stage stratified cluster random sampling method was used to recruit representative participants. In the first stage, 12 areas including urban or rural areas, Qinhuai, Jianye, Quanshan, Tongshan, Suining, Taicang, Changshu, Jiangyin, Jurong, Haimen, Sihong, Dafeng, which represented the overall geographical and economic situation in whole province, were sampled as the survey points by using systematic sampling. From each of these survey points, three streets / townships were randomly selected. In each street / township, two villages / neighborhoods were further randomly selected. All members in the households were invited to take part in the study. Participants aged ≥18 years were evaluated, of which 83 (4.8%) individuals were excluded from the analysis due to incomplete food frequency questionnaire (FFQ) survey data or abnormal caloric intakes (i.e., < 800 or > 5000 kcal/d), leaving us with data of 1739 individuals (53.8% female, *n* = 935) to be included in this study.

### Sociodemographic and lifestyle survey

Sociodemographic data such as age, gender, education level (illiterate, primary, secondary and senior secondary and above), job (low physical work, middle physical work, high physical work and other work) and economic status (low-income, middle-income, high-income and others) were collected by investigators who were trained and followed the same questionnaire instructions. Smoking / Drinking status was defined as who had smoking or drinking habits during the investigation [15].

### Anthropometric measurement

The anthropometric indices of participants with light clothing and without shoes were measured by well-trained examiners in a comfortable environment. Weight was measured to the nearest 10th of a kilogram. Height was measured to the nearest 10th of a centimeter with a stadiometer. All measurements were performed twice using a standard protocol and techniques [16]. Body mass index (BMI) was calculated as weight in kilograms divided by height in squared meters. BMI was classified into underweight, < 18.5 kg/m^2^; normal, ≥18.5 to < 24 kg/m^2^; overweight, ≥ 24 to < 28 kg/m^2^; obese, ≥28 kg/m^2^ [17].

### Dietary assessment

The data of food consumption was collected by validated and standardized food frequency questionnaire (FFQ) [18]. The FFQ contained hundreds of kinds of food, which basically covered dietary intake of residents in Jiangsu province for 1 year. Then we combined those kinds of food into 30 categories combined with food types. According to the dietary guidelines for Chinese residents [19], it was further merged into 19 food items, which represented typical Chinese diet (Table 1). Portion size for each food was established by using food models. Participants were asked to recall the frequency of consumption of individual food items (number of times per day, per week, per month and per year) and the estimated portion size. Food intake was converted into g / week for data analysis.

**Table 1:** Food groupings used in factor analysis

Food group	Example of food items
Cereals and tubers products	Rice, noodles, pasta, plain bread
Whole grains	Corn, barley, buckwheat
Dark-colored vegetables	Spinach, canola, carrot, spinach
Light-colored vegetables	Chinese cabbage, potato, onion
Pickled vegetables	Preserved vegetables, vegetables in soy sauce
Poultry	Chicken, duck meat
Red meats and its products	Pork, beef, lamb and those products
Eggs and eggs products	Whole eggs, yolk, white, preserved eggs
Fruit	Fresh and canned (no added sugar) fruits
Condiment	Sauce, vinegar, salt, monosodium glutamate
Oils	Colza oil, soybean oil, peanut oil
Soy products	Dried beans, beans flour, roasted broad bean
Milk and its products	Whole milk, skim milk, flavored milk, cheese, yogurt
Seafood	Fresh fish, dried fish, shellfish, shrimp
Nuts and seeds	Sesame, sunflower, peanuts, walnuts, almonds, hazelnuts, pine-nuts
Drink	Fruit or flavored drinks, fruit juice, soft drinks
Wine	Beer, rice wine, white wine
Pastry snacks	Cakes, pancake, mooncake
Other food	Fast food, canned food

### Preliminary exploration of dietary patterns

Dietary patterns were identified by using exploratory factor analysis (EFA) [20]. The Kaiser-Meyer-Olkin (KMO) measure of sample adequacy and Bartlett test of sphericity were used to assess data adequacy by factor analysis. Factor scores were orthogonally (varimax) rotated to minimize the correlation between factors and to improve the interpretability of factors. The result of KMO were 0.718 in men and 0.769 in women, respectively. Besides, the Bartlett test was significant both in men and women (*P* < 0.001). The result of KMO and Bartlett test indicated that the diet data would be useful. Orthogonal rotation with the Kaiser criterion (eigenvalues > 1.25) was used to determine the number of factors. Scores for each pattern were calculated as the sum of the products of the factor loading coefficient and the standardized intake of each food associated with that pattern. Food groups with factor loadings > |0.30| were included into the analysis (the information of the classification of food items was shown in Table 2), which represented that the foods had strongest relationship with the identified factor [20]. Dietary patterns were named according to the highest factor loading and interpretability. Factor scores were divided into four quartiles based on their distribution in each stratum.

**Table 2:** Factor loadings for 3 dietary patterns derived from factor analysis by gender^a^

Food groups	Male			Female		
	Pattern I	Pattern II	Pattern III	Pattern I	Pattern II	Pattern III
Poultry	0.687			0.483		
Light-colored vegetables	0.686			0.593		
Red meat and its products	0.546	0.471		0.528	0.327	
Cereals and tubers products	0.545			0.540		
Condiment	0.543		0.472	0.383		
Oils	0.397		0.385			
Dark-colored vegetables	0.350	0.536				
Eggs and eggs products		0.533			0.471	
Fruit		0.431	0.430	0.370	0.419	
Pickled vegetables		0.347				
Whole grains		0.336				
Drink						0.430
Soy products						
Milk and its products					0.399	
Other food					0.481	
Seafood					0.461	
Pastry snacks			0.580		0.673	
Nut			0.525			0.732
Wine			0.362			0.685

^a^Factor loading values < 0.30 were excluded for simplicity

### Statistical analysis

Categorical variables were presented as a number or percentage, while continuous variables were presented as mean ± standard deviation (SD). Chi-squared test was used to compare the differences in the characteristics of the participants of categorical data. T tests were used to compare continuous variables. The multivariable logistic regression analysis and the odds ratios (OR) and 95% confidence intervals (CIs) were calculated in order to estimate the associations between dietary patterns and overweight and obesity.

SEM was utilized to assess the association between overweight and obesity and its potential correlated factors by using the maximum likelihood estimation (MLE). SEM is an extension of the general linear model. It is mainly used to study the observation and latent variables at the same time. And it is one of the latest methods for examining the direct and indirect effects of a set of variables on outcomes. Through this method, it is possible to test the acceptability of the theoretical models in specific factors. Generally, it combines with two parts: the model of measurement [confirmatory factor analysis (CFA)] and the structural model (path analysis, generalization of regression analysis). CFA was used to test the fitting degree of dietary pattern detected by EFA. Then, SEM was utilized to test the conceptual model in gender groups. The SEM in this study includes exogenous latent variables (the dietary patterns from factor analysis and personal information of participants), external observation variables (all kinds of food groups in the dietary pattern), endogenous latent variables (participants with overweight and obesity) and adjustment variables (energy intake, smoking behavior, job, age group, education and economic status).

To confirm the model fit, the goodness-of-fit index (GFI), adjusted goodness-of-fit index (AGFI) and comparative fit index (CFI) equal to or greater than 0.90, parsimony goodness-of-fit index (PGFI) equals to or greater than 0.50 and root mean square error of approximation (RMSEA) equals to or less than 0.08 were applied [21]. Data management and statistical analysis were performed using IBM SPSS Statistics software Version 26.0 and AMOS Version 20.0. In all analyses, *P* values < 0.05 were considered significant.

## Results

### Basic information of participants

Table 3 revealed the descriptive statistics of participants according to whether they were overweight and obese or not. Overall, 1739 individuals (53.8% female, *n* = 935) with complete information were included in the analysis. The average of body weight, height and energy intake in men were significantly higher than that in women (*P* < 0.05). The average of BMI was 24.2 ± 3.2 and 24.1 ± 3.5 kg/m^2^ in men and women, respectively (*P* < 0.001). Overweight and obesity was found in 49.1% of individuals were overweight and obese, and there was no difference between men and women (51.2% of men and 47.2% of women, respectively; *P* = 0.090). Among participants, 52.2% (*n* = 420) men had smoking behavior, which was significantly higher than women (1.6% women had smoking behavior) (*P* < 0.001).

**Table 3:** Characteristics of study participants stratified by gender from Jiangsu Province, China in 2007–2014

Groups	Male		Female		t	*P* value
	Mean	SD	Mean	SD		
Body Weight (kg)	67.6	10.4	58.8	9.6	18.189	< 0.001
Height (cm)	167.1	6.5	156.0	5.9	37.303	< 0.001
Body mass index (kg/m^2^)	24.2	3.2	24.1	3.5	0.116	0.907
Energy intake (kcal/d)	2228.6	875.8	2133.7	807.7	2.355	0.020
	n	%	n	%	χ^2^	*P* value
Age group (years)					0.218	0.640
25 ~ 39	88	10.9	109	11.7		
40~	716	89.1	826	88.3		
Education level					86.083	< 0.001
Illiterate	62	23.5	220	16.2		
Primary	274	33.2	310	33.6		
Secondary	342	32.2	301	37.0		
Senior secondary or above	126	11.1	104	13.2		
Job					21.310	< 0.001
Low physical work	376	46.8	484	51.8		
Middle physical work	62	7.7	33	3.5		
High physical work	175	21.8	234	25.0		
Other physical work	191	23.8	184	19.7		
Economic status					1.36	0.715
Low-income	242	30.1	305	32.6		
Middle-income	350	43.5	395	42.2		
High-income	174	21.6	195	20.9		
Other-income	38	4.7	40	4.3		
Smoking behavior					590.873	< 0.001
No	384	47.8	920	98.4		
Yes	420	52.2	15	1.6		
Central obesity					0.231	0.631
No	512	63.7	585	62.6		
Yes	292	36.3	350	37.4		
Overweight and obesity					2.877	0.090
No	392	48.8	494	52.8		
Yes	412	51.2	441	47.2		

### Identification of dietary patterns

The factor loading matrix about dietary patterns using factor analysis was shown in Table 2. Eigenvalues, the scree plot test and interpretability were evaluated to explain the food items.

**Fig. 1:**
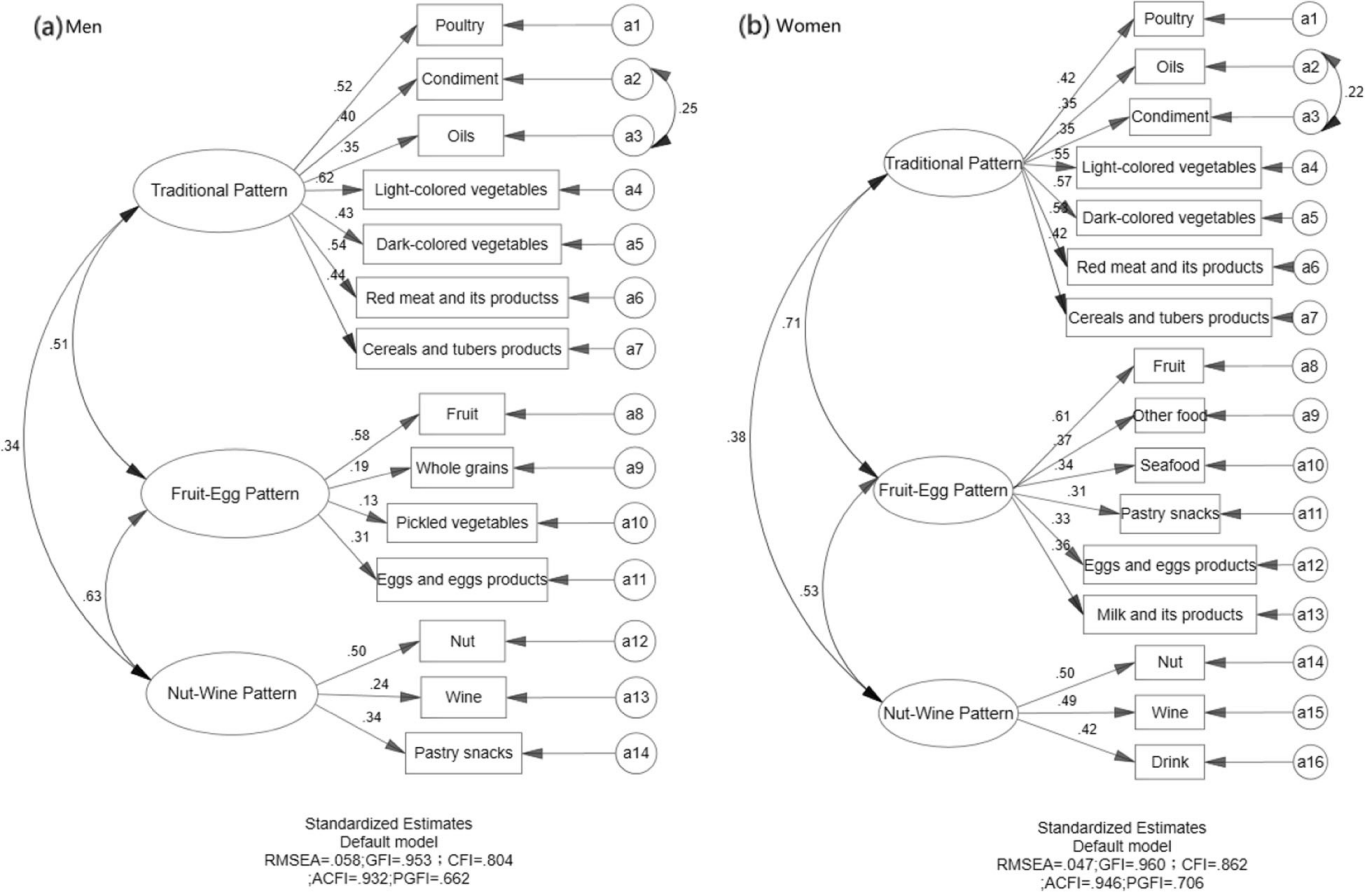
Measurement models of the latent construct of three dietary patterns among adults from Jiangsu Province, China in 2007–2014 (**a** men and **b** women). In model of men: RMSEA = 0.063, GFI = 0.946, CFI = 0.763, ACFI = 0.923 and PGFI = 0.667. In model of women: RMSEA = 0.047, GFI = 0.960, CFI = 0.862, ACFI = 0.946 and PGFI = 0.706. *Rectangles* indicate observed variables, and *oval* is latent variable in the model. All factor loadings and regression coefficients in the figure are significant (*P* < 0.05)

Among men, three dietary patterns were finally established, namely ‘traditional dietary pattern’ (pattern I), ‘fruit-egg dietary pattern’ (pattern II), ‘nut-wine dietary pattern’ (pattern III). The explained variance with pattern I (Eigenvalue = 2.869), pattern II (Eigenvalue = 1.478) and pattern III (Eigenvalue = 1.478) mentioned was 15.102, 7.779, and 6.651%, respectively. Then, we put food groupings in three dietary patterns with higher factor loadings into confirmatory factor analysis model (Fig. 1a). Ultimately, the traditional dietary pattern was loaded heavily on poultry, light-colored vegetables, red meat and its products, cereals and tubers products, condiment, oils and dark-colored vegetables. The fruit-egg dietary pattern was loaded heavily on fruit, whole grains, pickled vegetables and eggs and eggs products. The nut-wine dietary pattern was loaded heavily on nut, wine and pastry snacks.

Among women, similarly, three dietary patterns were detected by factor analysis. After considering the factor loadings, interpretability and the association with the dietary patterns mentioned by men above, the three dietary patterns obtained by women were named as ‘traditional dietary pattern’ (pattern I), ‘fruit-egg dietary pattern’ (pattern II), ‘nut-wine dietary pattern’ (pattern III). The explained variance with pattern I (Eigenvalue = 3.185), pattern II (Eigenvalue = 1.364) and pattern III (Eigenvalue = 1.287) mentioned was 16.765, 7.180, and 6.775%, respectively. CFA was used to make further efforts to confirm the food groupings in the dietary patterns (Fig. 1b). Finally, the traditional dietary pattern was loaded heavily on poultry, oils, condiment, light-colored vegetables, dark-colored vegetables, red meat and its products, cereals and tubers products. The fruit-egg dietary pattern was loaded heavily on fruit, other food, seafood, pastry snacks, eggs and eggs products and milk and its products. The nut-wine dietary pattern was loaded heavily on nut, wine and drink.

### Association between dietary patterns and overweight and obesity using multivariate logistic regression

Multivariate logistic regression model displayed in Table 4 was used to analyze the relationship between dietary patterns and overweight and obesity. The result showed that high intake of traditional pattern increased the risk of overweight and obesity in men (composed to Q1, Q2 ~ Q4 OR = 1.560, 1.638 and 1.954; 95%CI: 1.039 ~ 2.351, 1.076 ~ 2.495 and 1.258 ~ 3.036, respectively, *P* < 0.05). However, the traditional dietary pattern in women was not associated with overweight and obesity. Additionally, men with highest quartile of the fruit-egg dietary pattern in unadjusted model had a positive association with overweight and obesity (composed to Q1, OR = 1.695; 95%CI: 1.139 ~ 2.521, *P* < 0.05). By contrast, women in the highest quartile of fruit-egg dietary pattern had a negative association with overweight and obesity (composed to Q1, OR = 0.651; 95%CI: 0.452 ~ 0.937, *P* < 0.05). Nevertheless, the nut-wine dietary pattern was not significantly linked with overweight and obesity in either gender (*P* > 0.05).

**Table 4:** Odds ratios (95% confidence intervals) for overweight and obesity across quartiles of dietary patterns

Group	Dietary pattern	Model 1^a^			Model 2^b^		
Men	Traditional pattern	OR	95%CI	*P*	OR	95%CI	*P*
	Q1	1.000			1.000		
	Q2	**1.689**	**(1.137 ~ 2.509)**	**0.009**	**1.563**	**(1.039 ~ 2.351)**	**0.032**
	Q3	**1.758**	**(1.183 ~ 2.612)**	**0.005**	**1.638**	**(1.076 ~ 2.495)**	**0.021**
	Q4	**2.336**	**(1.566 ~ 3.484)**	**< 0.001**	**1.954**	**(1.258 ~ 3.036)**	**0.003**
	Fruit-egg pattern						
	Q1	1.000			1.000		
	Q2	0.786	(0.531 ~ 1.165)	0.230	0.825	(0.544 ~ 1.249)	0.363
	Q3	1.041	(0.704 ~ 1.539)	0.842	0.922	(0.603 ~ 1.410)	0.708
	Q4	**1.695**	**(1.139 ~ 2.521)**	**0.009**	1.515	(0.984 ~ 2.332)	0.059
	Nut-wine pattern						
	Q1	1.000			1.000		
	Q2	1.062	(0.718 ~ 1.570)	0.765	1.193	(0.787 ~ 1.808)	0.407
	Q3	1.020	(0.690 ~ 1.508)	0.921	1.071	(0.706 ~ 1.625)	0.748
	Q4	1.377	(0.930 ~ 2.040)	0.110	1.203	(0.785 ~ 1.842)	0.396
Women	Traditional pattern						
	Q1	1.000			1.000		
	Q2	1.147	(0.798 ~ 1.649)	0.459	1.228	(0.841 ~ 1.792)	0.288
	Q3	1.008	(0.701 ~ 1.450)	0.966	1.190	(0.807 ~ 1.754)	0.379
	Q4	1.017	(0.707 ~ 1.463)	0.926	1.114	(0.759 ~ 1.636)	0.581
	Fruit-egg pattern						
	Q1	1.000			1.000		
	Q2	0.835	(0.581 ~ 1.201)	0.332	0.915	(0.621 ~ 1.348)	0.653
	Q3	0.722	(0.502 ~ 1.039)	0.079	0.879	(0.594 ~ 1.300)	0.519
	Q4	**0.651**	**(0.452 ~ 0.937)**	**0.021**	0.798	(0.542 ~ 1.173)	0.251
	Nut-wine pattern						
	Q1	1.000			1.000		
	Q2	1.120	(0.778 ~ 1.613)	0.543	1.057	(0.722 ~ 1.549)	0.774
	Q3	1.423	(0.988 ~ 2.049)	0.058	1.308	(0.890 ~ 1.921)	0.171
	Q4	1.082	(0.751 ~ 1.559)	0.672	0.985	(0.668 ~ 1.452)	0.937

^a^Model 1: unadjusted model

^b^Model 2: adjusted energy intake, age group, education level, job, smoking and income status

### Structural model

**Fig. 2:**
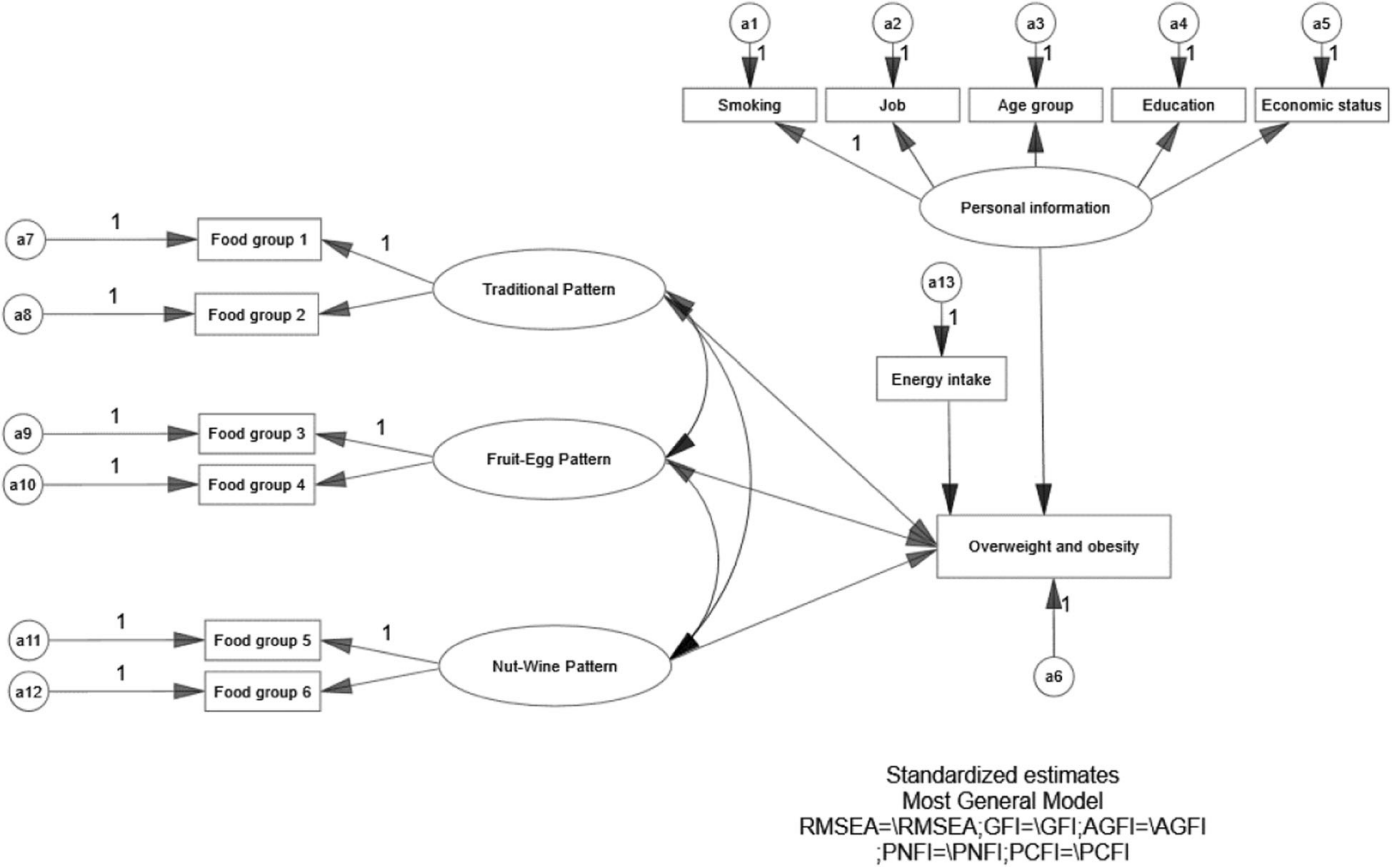
A conceptual SEM model for the association of socio-demographic, dietary pattern, and life style with overweight and obesity

**Fig. 3:**
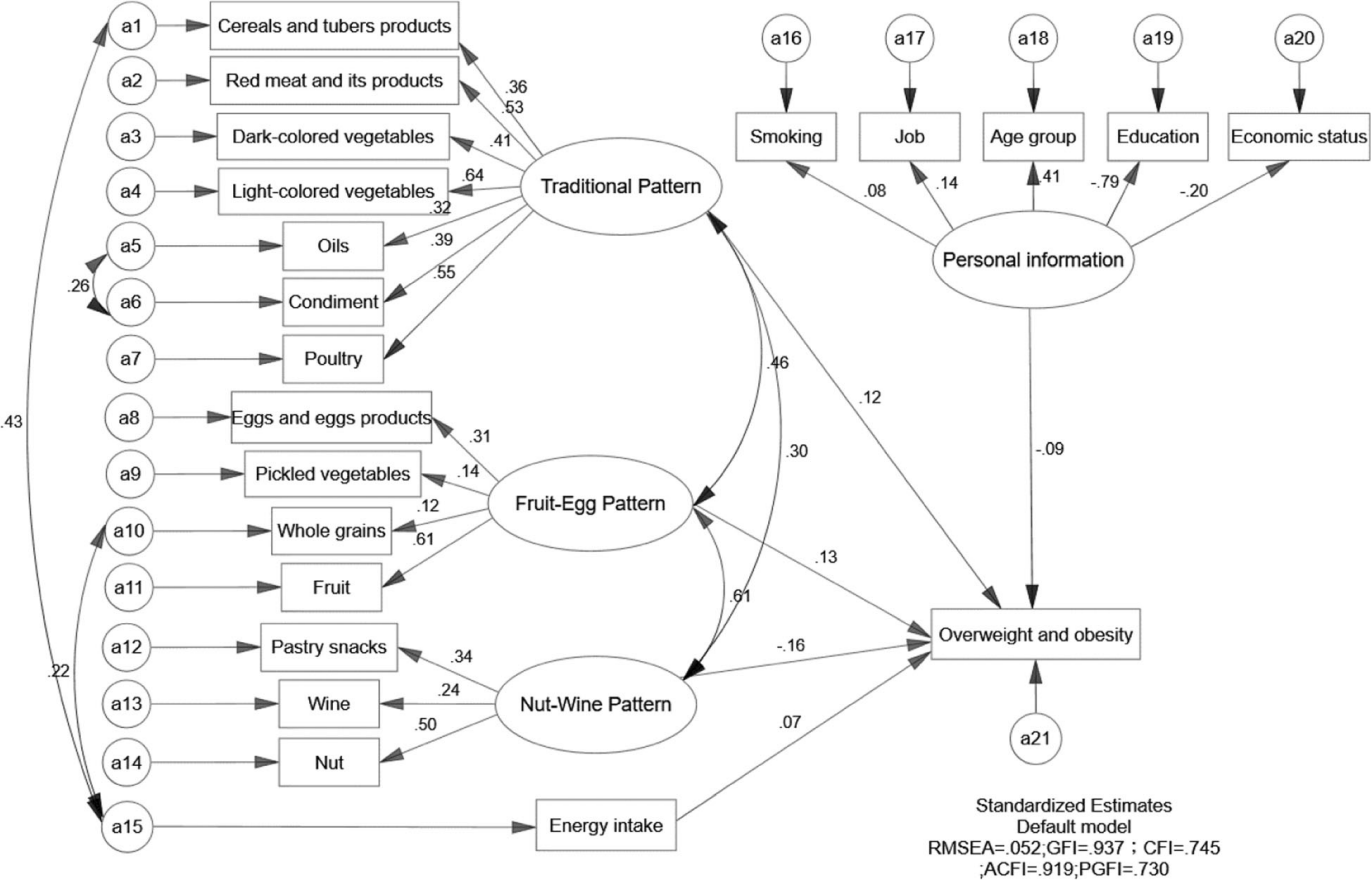
Final structural models in men. The path standardized coefficients of variables are presented on pathways. RMSEA = 0.052, GFI =0.937, CFI = 0.745, ACFI = 0.919, PGFI = 0.730 and. a error

Figure 2 showed the SEM diagram with standardized estimates for the relationships between overweight and obesity, dietary patterns and risk factors. The one-sided arrows from three dietary patterns, energy intake and personal information to overweight and obesity represent the regression coefficients; whereas the arrows from the dietary patterns latent variable and personal information mediation variable to food groups, smoking behavior, job, education level, age group and economic status, respectively, indicating the standardized factor loadings of the measured variables. The two-sided arrows represent the correlation coefficients between dietary patterns. The result of parameter estimates from the structural equation modelling of dietary patterns and overweight and obesity among individuals shown as Table 5 and Fig. 3. Only traditional dietary pattern in men had positive association with overweight and obesity (*P* < 0.05).

**Table 5:** Parameter estimates from the structural equation modelling of dietary patterns and overweight and obesity among individuals from Jiangsu Province, China in 2007–2014

Path analysis	Groups	Non-standardized coefficient	Standardized coefficients	S.E.	C.R.	*P*
Traditional pattern → Overweight and obesity	Men	0.001	0.121	0.000	2.083	**0.037**
	Women	0.004	0.089	0.003	1.157	0.247
Fruit-egg pattern → Overweight and obesity	Men	0.007	0.133	0.006	1.200	0.230
	Women	−0.006	−0.118	0.005	−1.231	0.218
Nut-wine pattern → Overweight and obesity	Men	−0.010	−0.156	0.007	−1.448	0.148
	Women	0.000	−0.004	0.005	−0.064	0.949

The final SEM model was obtained by increasing residual correlations and modification indices. The goodness-of-fit indices of the final model indicated an acceptable fit (the model of men: RMSEA = 0.052, GFI = 0.937, CFI = 0.745, ACFI = 0.919, PGFI = 0.730). Traditional dietary pattern had a positive effect on the overweight and obesity (β = 0.121, *P* < 0.05).

## Discussion

The health of residents has always been a key issue of social concern. Overweight and obesity due to overnutrition have transformed from small health problems in developed countries to major health problems affecting global public health issue. It is believed that dietary patterns play an important role in the development of overweight and obesity [22–24]. In this prospective study, three distinct dietary patterns were identified: traditional dietary pattern, fruit-egg dietary pattern and nut-wine dietary pattern using EFA and CFA [25]. SEM and multivariate logistic regression analysis were used to explore the relationship between these dietary patterns and overweight and obesity in Jiangsu Province. Finally, the combined results of SEM and multivariate logistic regression showed that the traditional dietary pattern had a greater risk of overweight and obesity only in men. However, both the fruit-egg dietary pattern and the nut-wine dietary pattern were not significantly correlated with overweight and obesity.

The traditional dietary pattern composing with poultry, light-colored vegetables, red meat and its products, cereals and tubers products, condiment, oils and dark-colored vegetables represented a typical and traditional diet structure in Jiangsu Province of China. In the present study, the relationship between the traditional dietary pattern and later overweight and obesity in China is relatively vague [11, 26, 27]. In this study, we found that traditional dietary pattern was positively linked to later overweight and obesity in men. The result was similar to a study in Shanghai Food Consumption Survey (SHFCS), which found that people who followed a rice staple pattern composing rice, starchy roots and tubers, vegetables, pork, poultry, organ meats and processed meats had a risk of general obesity [26]. A main characteristic of the traditional dietary pattern in men is the high intake of cereals and tubers products. In our study, cereals and tubers products and whole grains were separated to highlight the effect of refined grains on later overweight and obesity. As we all know, refined grains are major source of dietary carbohydrate. A birth cohort about 918 mother-singleton child dyads showed that refined-grain intake during pregnancy was positively linked with the risk of overweight and obesity at 7 age. And using one serving of whole grains per day instead of refined grains during pregnancy can reduce the risk of overweight and obesity in offspring by 10% [28]. High glycemic index carbohydrates cause rapid changes in blood glucose and insulin levels, similar to the pharmacokinetics of addictive substances. Sugar causes addictive cravings, glucose and insulin signal the midbrain limbic system to change dopamine levels. These characteristics make carbohydrate with high glycemic index become the reasonable inducement of food addiction [29]. The evidence that high carbohydrate from refined grains associated with obesity has also been confirmed in previous studies [30–32]. Carbohydrate quality index (CQI) was based on four criteria: crude fiber intake, dietary glycemic index (DGI), whole grains / total grains ratio and solid carbohydrates / total carbohydrates ratio. A cross-sectional study indicated that higher CQI was negatively associated with the prevalence of obesity [33]. Hence, it could be refined grains, not whole grains, that causes the prevalence of obesity in adults in Jiangsu Province of China. Another main characteristic of this pattern is the high consumption of red meat and its products. Red meat mainly refers to pork, beef and other meat that appears red before cooking [34]. Red meat and its products contain a lot of fat, and are known as energy-dense food. Excessive consumption of red meats has been confirmed to be positively correlated with overweight and obesity through studies across different populations [35, 36]. A cross-sectional study figured out that high consumption of red meat (≥ 81.5 g/d) and saturated fatty acid from red meat consumption (≥ 4.3 g/d) had higher correlation with the prevalence of central obesity, hypertriglyceridemia and metabolic syndrome [37]. In addition, a study from China Health and Nutrition Survey indicated that higher intake of fatty fresh red meat was the risk of central obesity both in men and women and leaded to higher waist circumference only in men [38–40]. The combination of high intake of carbohydrates and fats might be more likely to cause later overweight and obesity [39]. The western dietary pattern known as the representative of high-fat and high-carbohydrate diet has been proved to be positively associated with overweight and obesity [27, 41, 42]. The global prevalence of overweight obesity has tripled between 1980 and 2014 as the global impact of Western dietary patterns continues to increase [43].

Many healthy dietary patterns, such as Mediterranean diet [44–46] and DASH diet [47–49], show that vegetable intake can reduce the prevalence of overweight and obesity. Notwithstanding, the traditional dietary pattern in our study is counterintuitive, as it is more analogue to the DASH diet [49] and Mediterranean diet [50] about abundant consumption of fresh vegetables. But this resemblance is more just a representation of similarity. One of the most important reasons is that condiments and oils are rarely considered when including vegetables in food grouping. Fresh vegetables in the Mediterranean diet and DASH diets are generally presented in the form of salads without cooking. This is different from other parts of the world, such as South Korea [51] and Japan [52] where consume high salted vegetables resulting in high intake of sodium. In the traditional Chinese diet, oil and condiments such as salt are usually added to vegetables to increase the taste. Among many condiments, the consumption of salt, which is often used to improve the taste of foods and added in other condiments, is the most frequently consumed. Salt is acknowledged as an independent risk factor for hypertension [53, 54]. A study reported that the intake of salt was associated with overweight and obesity and put forward a hypothesis that abundant salt intake might be an independent risk factor for overweight and obesity [55]. Increasing intake of salt can cause thirst response. At the same time, with the increase of types and taste of sugar-sweetened beverages, increasing number of people will choose to drink sugar-sweetened beverages instead of water to quench thirst. This closed-loop process could lead to an increase in overweight and obesity rates [56, 57]. A study based on Australian National Children’s Nutrition and Physical Activity Survey including 4283 participants indicated that each additional 1 g/d of salt was associated with a 17 g/d greater intake of sugar-sweetened beverages. With the increasing intake of sugar-sweetened beverages, the prevalence of overweight and weight increased [58]. In addition, oil intake might play a positive role in overweight and obesity in adult. When oil such as rapeseed oil was fried at 180 °C and 220 °C, the contents of trans fatty acid and saturated fatty acid increased significantly, whereas the content of cis unsaturated fatty acid decreased (*P* < 0.001) [59]. The increase of trans fatty acids and saturated fatty acids will undoubtedly raise the possibility of overweight and obesity [60, 61]. Moreover, other condiments such as dietary sugar [62], monosodium glutamate [63] also displayed positive correlation with overweight and obesity in adult. The use of oil and condiments in the diet is in line with the traditional Chinese dietary habits. Therefore, the role of oils, condiments as well as cooking methods should be taken into account in dietary analysis. However, the hypothesis above cannot fully explain the relationship between this dietary pattern and overweight and obesity. Because the traditional dietary pattern only showed a positive correlation with overweight and obesity in men, but not in women in our study. This phenomenon could be attributed to the high smoking prevalence of men [64]. In our study, the prevalence of current smoker for men is 52.2%, which was significantly higher than women (1.6% women had smoking behavior) (*P* < 0.001). A study of 5 countries including 89,432 reported that an increase of 100 g of fruits and vegetables per day was associated with a slightly lower risk of weight gain. Compared with non-smokers, stable smokers had a significantly lower risk of weight loss, whereas started smokers had higher risk of weight loss [65]. Smoking behavior might reduce the impact of vegetables on overweight and obesity in men.

Fruit-Egg dietary pattern and Nut-Wine were two additional dietary patterns that we found, which had no association with overweight and obesity both in men and women. The non-significant relationship might be the result of the interaction of some healthy food groups and unhealthy food groupings. On the one hand, the adverse food items, such as pickled vegetables, pastry snacks and wine were recognized to be associated with a higher risk of obesity. In order to better present the dietary habits of Jiangsu Province, pickled vegetables and fresh vegetables were actually counted separately as two separate food groupings in this study. Excessive nitrite and salt in pickled vegetables and excess content of sugar and energy contents in pastry snacks might be the cause of high body mass index [62, 66, 67]. Besides, the relationship between alcohol intake and obesity is more ambiguous. A review about obesity and alcohol consumption suggested that light-to-moderate alcohol consumption had no association with the development of obesity, while excessive alcohol use was positively linked to obesity [68]. On the other hand, fruits, seafood, milk and nuts, etc., considered the healthy constituent in the pattern, would counteract the unhealthy effect on overweight and obesity in men and women [69–72]. Although these two patterns did not increase the risk of obesity, we should also be concerned about the adverse components of the dietary pattern. For instance, Higher consumption of pickled vegetables is still frequent in Jiangsu, especially in rural areas, and more attention should be paid to the health risks of pickled vegetables. Recently, the intake of snacks is increasing, which will undoubtedly shift the traditional Chinese diet of three meals a day to three meals plus snacks. Therefore, more studies should be taken into account to find the health effects of this change. In addition, I think it is more important to pay attention to the amount of alcohol and to people who usually do not drink but will binge drink in some situations.

To the best of our knowledge, this was the first time that gender-specific pathways of associations of socio-behavioral factors with overweight and obesity were tested in the observational population-based prospective nutrition and heavy study using SEM and multivariate logistic regression in Jiangsu Province of China. Second, the sampling was based on a representative population including different socio-demography and geography. However, the present investigation also had some weaknesses. Firstly, it was limited by its cross-sectional design, which hindered any causal relationship. Secondly, other confounders that might have impacts on overweight and obesity, such as physical activity, sleeping time, etc., were not considered in the present study, which might have some slight effects on our results. Thirdly, FFQ exists recall bias in the process of investigation.

## Conclusions

Three dietary patterns were obtained through EFA and CFA both in men and women in this study: the traditional dietary pattern, the fruit-egg dietary pattern and the nut-wine dietary pattern. The traditional dietary pattern had a positive association with overweight and obesity only in men. The fruit-egg dietary pattern and nut-wine dietary pattern were not associated with overweight and obesity both in men and women.

## Data Availability

The datasets used and/or analyzed during the current study are available from the corresponding author on reasonable request.
